# The genetic polymorphisms of immune‐related genes contribute to the susceptibility and survival of lymphoma

**DOI:** 10.1002/cam4.6131

**Published:** 2023-06-16

**Authors:** Chaoyang Gu, Can Can, Jinting Liu, Yihong Wei, Xinyu Yang, Xiaodong Guo, Ruiqing Wang, Wenbo Jia, Wancheng Liu, Daoxin Ma

**Affiliations:** ^1^ Department of Hematology Qilu Hospital of Shandong University Jinan China

**Keywords:** disease progression, genetic polymorphisms, immune‐related genes, lymphoma, survival

## Abstract

**Background:**

Though immunological abnormalities have been proven involved in the pathogenesis of lymphoma, the underlying mechanism remains unclear.

**Methods:**

We investigated 25 single nucleotide polymorphisms (SNPs) of 21 immune‐related genes and explored their roles in lymphoma. The genotyping assay of the selected SNPs was used by the Massarray platform. Logistic regression and Cox proportional hazards models were used to analyze the associations of SNPs and the susceptibility of lymphoma or clinical characteristics of lymphoma patients. In addition, Least Absolute Shrinkage and Selection Operator regression was used to further analyze the relationships with the survival of lymphoma patients and candidate SNPs, and the significant difference between genotypes was verified by the expression of RNA.

**Results:**

By comparing 245 lymphoma patients with 213 healthy controls, we found eight important SNPs related to the susceptibility of lymphoma, which were involved in JAK–STAT, NF‐κB and other functional pathways. We further analyzed the relationships between SNPs and clinical characteristics. Our results showed that both IL6R (rs2228145) and STAT5B (rs6503691) significantly contributed to the Ann Arbor stages of lymphoma. And the STAT3 (rs744166), IL2 (rs2069762), IL10 (rs1800871), and PARP1 (rs907187) manifested a significant relationship with the peripheral blood counts in lymphoma patients. More importantly, the IFNG (rs2069718) and IL12A (rs6887695) were associated with the overall survival (OS) of lymphoma patients remarkably, and the adverse effects of GC genotypes could not be offset by Bonferroni correction for multiple comparison in rs6887695 especially. Moreover, we determined that the mRNA expression levels of IFNG and IL12A were significantly decreased in patients with shorter‐OS genotypes.

**Conclusions:**

We used multiple methods of analysis to predict the correlations between lymphoma susceptibility, clinical characteristics or OS with SNPs. Our findings reveal that immune‐related genetic polymorphisms contribute to the prognosis and treatment of lymphoma, which may serve as promising predictive targets.

## 1 INTRODUCTION

Lymphoma is a heterogeneous and complex group of lymphocytic neoplasms, which is accompanied by a broad range of immune regulatory disorder phenotypes. Although great progress has been obtained using chemotherapy and targeted approaches over recent decades, the overall treatment efficacy of lymphoma is still disappointing. Therefore, it is of vital importance to explore its detailed mechanism and find novel therapeutic targets for lymphoma.

Abnormal immune mechanism is considered critical in the progress of lymphoma.^1^ Immune dysfunction exists in lymphoma patients, and T cells, as the most important component of the immune system, are found to be numerically and functionally defective.^2^, ^3^, ^4^ Our previous results also showed that circulating Th17 immune response in lymphoma patients may be associated with the response to treatment and with different stages of diseases.^5^ Furthermore, targeting the immune molecules has been shown to be effective against lymphoma.^4^, ^6^ Therefore, further researches on pathological mechanism in the function of the immune system are required.

It has been reported that there were various identified pathogenic mutations in immune‐related genes, which were involved in BCR and TLR signaling, NF‐κB pathway, cell apoptosis, and other functional pathways.^7^ Moreover, many other genetic polymorphisms of immune‐related genes were reported to be closely related to the susceptibility and risk of lymphoma. Butterbach et al. found that polymorphisms of nine genes including IFNG, IL12A, and STAT family were significantly associated with the risk of lymphoma.^8^ A polymorphism in the interferon regulatory factor 4 gene, rs12211228, has a significant association with NHL risk.^9^ And the gene polymorphisms of IL‐10 (rs1800890, rs1800896, and rs1800872) and IL‐18 (rs1946518) were also associated with the risk or susceptibility of lymphoma.^1^, ^10^ Patients with AA genotype in rs1585215 of NFKB1 in the NF‐κB pathway were more likely to develop Stage IV and extranodal lesions when diagnosed. Besides, AG genotype of TNF‐α was more likely to develop EBV‐related chronic lymphocytic leukemia than homozygous GG.^11^ As one of the genes that control the activity of T lymphocytes, protein tyrosine phosphatase nonreceptor 22 (PTPN22) has also been reported to play an important role in the development of many autoimmune diseases in recent years. PTPN22 dephosphorylates NLRP3 inflammatory cells to limit the retention of NLRP3 in autophagy and to degrade it in autolysosomes,^12^ and its rs2476601 genotype can down‐regulate the TCR signal system and reduce the affinity of Csk and T cells.^13^ In addition, Integrin Alpha M (ITGAM) and Poly ADP ribose polymerase‐1 (PARP1) were also proved to be a new prognostic marker in diffuse large B‐cell lymphoma (DLBCL)^14^ and mantle cell lymphoma^15^ Moreover, we have previously reported the positive associations between NLRP3 inflammasome associated polymorphisms and certain hematological diseases in the hematopoietic system, including lymphoma.^1^

To deeply discover new markers related to the etiology, progression and prognosis of lymphoma, we used the Agena Massarray technology platform to detect 25 candidate SNPs of 21 genes (TNFA, IFNG, IFNL3, IL2, IL4, IL6R, IL9, IL10, IL12A, IL22, TGFB1, TBX21/TBET, STAT1, STAT3, STAT5B, STAT6, GATA3, NFKBIA, ITGAM, PTPN22, PARP1) in 245 lymphoma patients and 213 healthy controls.^8^, ^10^, ^16^, ^17^, ^18^, ^19^, ^20^, ^21^, ^22^, ^23^, ^24^, ^25^, ^26^, ^27^, ^28^, ^29^, ^30^, ^31^, ^32^, ^33^, ^34^, ^35^, ^36^, ^37^, ^38^, ^39^, ^40^, ^41^, ^42^, ^43^, ^44^, ^45^, ^46^, ^47^, ^48^, ^49^, ^50^, ^51^, ^52^, ^53^, ^54^, ^55^, ^56^, ^57^ All the 25 candidate SNPs were based on previous research studies about immune‐related genetic polymorphisms on the susceptibility, prognosis, progression and stage of many cancers. And we confirmed them by SNP database, ClinVar or dbVar database that the genetic polymorphisms were associated with drug response, risk factors, and pathogenesis. Then we further analyzed the susceptibility of lymphoma and the relationship between SNPs and clinical characteristics. Our results will provide evidence for clinical diagnosis and management of lymphoma.

## 2 MATERIALS AND METHODS

### 2.1 Characteristics of the subjects

A total of 245 patients with lymphoma (131 males and 114 females) were included in this study from March 2008 to October 2021 at Qilu Hospital of Shandong University with a median age of 53 years (14–90 years). The diagnosis and classification of lymphoma were confirmed strictly according to the WHO (2016) classification and Ann Arbor stage.^58^ Meanwhile, 213 healthy controls (125 males and 88 females) participated in the study, with a median age of 51 years (16–92 years). The basic characteristics of all participants, such as age, gender, the peripheral blood count, lactate dehydrogenase (LDH), and stratification of lymphoma patients were shown in Table 1. Informed consent was obtained from all patients before enrollment in the study in accordance with the Declaration of Helsinki. This study was conducted with the approval of the institutional ethics committee (KYLL‐202204‐059).

**TABLE 1:** The demographic and clinical characteristics of lymphoma patients and healthy controls.

Parameters	Cases, *n* (%)	Controls, *n* (%)
Age		
Median(range)	53 (14–90)	51 (16–92)
>60	91 (37.14)	65 (30.52)
≤60	154 (62.86)	148 (69.48)
Sex		
Male	131 (53.47)	125 (58.69)
Female	114 (46.53)	88 (41.31)
WBC (×10^9^/L)		
Median(range)	6.07 (0.88–69.82)	NA
>15	16 (6.53)	NA
≤15	229 (93.47)	NA
PLT (×10^9^/L)		
Median(range)	231 (9–687)	NA
>250	100 (40.82)	NA
≤250	145 (59.18)	NA
HGB (g/L)		
Median(range)	125 (33–162)	NA
>110	174 (71.02)	NA
≤110	71 (28.98)	NA
LDH (U/L)		
Median(range)	239 (117–2493)	NA
>Normal	123 (52.34)	NA
≤Normal	112 (47.66)	NA
Subtype		
MBN	200 (81.63)	NA
Non‐MBN	45 (18.37)	NA
Ann Arbor stage		
I	21 (8.57)	NA
II	26 (10.61)	NA
III	88 (35.92)	NA
IV	110 (44.90)	NA

Abbreviations: HGB, hemoglobin; LDH, lactate dehydrogenase; MBN, Mature B‐Cell Neoplasms; NA, not applicable; Non‐MBN, Non‐mature B‐Cell Neoplasms; PLT, platelet count; WBC, white blood cell.

### 2.2 DNA extraction

Leukocytes of peripheral blood or bone marrow were collected from the participants involved in our study. For the same patient, the DNA and RNA were extracted from the same type of biological sample.^59^, ^60^, ^61^, ^62^ Genomic DNA was isolated by standard methods according to the manufacturer's instruction of TIANamp blood DNA kit (Tiangen Biotech). The quality and concentration of DNA were determined using a DeNovix DS‐11 Spectrophotometer (DeNovix Inc.).

### 2.3 The detection of genotyping

The selected functional SNPs or hot SNPs were all summarized and aligned through the dbSNP and the UCSC databases. Then Agena's Assay Designer 4.0 software was used to evaluate the primers which were designed for multiple SNP sites. Finally, the PAGE primer purification method was used to synthesize three primers which corresponded with each SNP site, two PCR primers and one UEP primer. The genomic DNA was applied for genotyping assay of the selected SNPs using the Massarray platform (Agena Bioscience). Approximately 20 ng of genomic DNA from each sample was used for genotyping. After the DNA samples were amplified via multiplex PCR, allele detection was performed through MALDI‐TOF mass spectrometry.

### 2.4 Reverse transcription and quantitative polymerase chain reaction (RT‐qPCR)

Total RNA from bone marrow or peripheral blood was extracted with TRIZOL™ reagent (REF: 15596018; Life Technologies) and detected by DeNovix DS‐11 Spectrophotometer. Then the RNA was reversely transcribed into cDNA using Evo M‐MLV RT Premix for qPCR (Code No. AG11706; Accurate Biotechnology Co., Ltd). Quantitative PCR (qPCR) was conducted by SYBR Green qPCR Mix (AH0104; SparkJade), and the relative gene expression was determined using a Roche LightCycler 480II system. All amplification primers and housekeeping gene β‐actin primers were designed by Primer Premier 5.0, and the sequences were as follows: IL‐12A forward: CTC CAG AAG GCC AGA CAA ACT C, IL‐12A reverse: GCC AGG CAA CTC CCA TTA GTT A; IFNG forward: AAG TGA TGG CTG AAC TGT CG, IFNG reverse: TAC TGG GAT GCT CTT CGA CC; β‐Actin forward: GAA GAG CTA CGA GCT GCC TGA; β‐actin reverse: CAG ACA GCA CTG TGT TGG CG. And the mRNA expression was analyzed by the comparative cycle threshold (Ct) method.

### 2.5 Statistical analysis

The chi‐square test was used to determine the difference in gender or age between the lymphoma patients and controls. Efficient implementations of exact tests were used to conduct the Hardy–Weinberg equilibrium of all 25 candidate SNPs. SNPs in HWE (*p* > 0.001) and minor allele frequency (MAF) >1% in the general population were included in the study. The relationships between SNPs phenotype or allele frequency and lymphoma susceptibility, blood routine indices, lymphoma stages, or survival analysis were determined in our study. Briefly, univariate binary logistic regression was used to analyze the differences between SNPs and susceptibility, peripheral blood count and LDH. Multivariate logistic regression analysis was used to analyze the relationship between Ann Arbor stage and lymphoma SNPs. All odds ratios (ORs) and corresponding 95% confidence intervals (95% CIs) were analyzed, and also adjusted for age and gender and corrected by Bonferroni to correct the analytic results. The purpose of Bonferroni correction in our study was to reject all the possibility of false positive results, and eliminate false positive results by the *p*‐value adjusted.

Cox proportional hazards model was used to analyze the relationship between the Overall survival (OS) of lymphoma patients and SNPs, and the hazard ratio (HR) and corresponding 95% CI were also adjusted and corrected by Bonferroni. The gene expression was calculated by the Mann–Whitney test. Adjusted two‐tailed *p*‐value < 0.05 was considered statistically significant. Efficient implementations of exact tests were performed by Plink software, and other statistical analyses were performed using IBM SPSS Statistics 22 software (SPSS Inc.) and R (version 4.1.2).

To further validate the accuracy of our results, the Least Absolute Shrinkage and Selection Operator (LASSO) regression was applied to feature selection and model construction. The “lasso” minimizes the residual sum of squares subject to the sum of the absolute value of the coefficients being less than a constant. Because of the nature of this constraint it tends to produce some coefficients that are exactly zero and hence gives interpretable models.^63^ In our study, the R‐package “glmnet” was used to select the most effective prognostic markers in 25 candidate SNPs and its corresponding regression coefficient by the penalty parameter λ with the least error through the ten‐fold cross‐validation. The “caret” package was used to get the 70% training data from all the SNPs data sets randomly, and each genetic model was run 1000 times to select the most accurate model. The performance of prediction model was evaluated by receiver operating characteristic (ROC) curves. Besides, the hazard ratios of the selected SNPs obtained from the LASSO regression model were calculated by univariate Cox analysis, and the results were also adjusted by age and gender. In addition, based on the 30% testing data predicted by LASSO regression model, we performed Wilcoxon analysis for the two survival states.

## 3 RESULTS

### 3.1 The characteristics of all participants

The characteristics of lymphoma patients and controls are shown in Table 1. There was no significant difference in the distribution of age (median 53, range 14–90 versus median 51, range 16–92) and gender (male/female: 131/114 vs. 125/88) between lymphoma patients and controls. Among 245 lymphoma patients, 15 were Hodgkin's Lymphoma and 230 were non‐Hodgkin's lymphoma, which including 200 mature B‐cell neoplasms and 30 other types of non‐Hodgkin's lymphoma. According to the criteria of Ann Arbor stage, the lymphoma group was further divided into four subgroups, including 21 cases at Stage I, 26 at Stage II, 88 at Stage III, and 110 at Stage IV. In this research, we calculated the Hardy–Weinberg equilibrium and minor allele frequency (MAF) and evaluated the applicability of all 25 candidate SNPs. Due to the low detection rate of rs2227491, the SNP was excluded to ensure the reliability of the result, and the remaining 24 SNP loci were used for the following analysis (Table 2).

**TABLE 2:** Selected genes and SNPs.

Gene	SNP	Variant	Variant allele	MAF	HWE (*p*‐value)	Variant type (SNP database)	Functional consequence (SNP database)	Annotation by SNP or ClinVar or dbVar database	References
TNFA	rs1800629	31,575,254 G > A	A	0.05679	0.6454	SNV	2KB_upstream_variant, Upstream_transcript_variant	Risk factor, uncertain‐significance, affects, drug‐response, pathogenic	16, 17
IFNG	rs2069718	68,156,382 A > G	G	0.1208	1	SNV	Intron_variant	Likely‐pathogenic	18
IFNL3	rs12979860	39,248,147 C > T	T	0.06071	0.6751	SNV	Intron_variant	Drug‐response, pathogenic, benign	19
IL2	rs2069762	122,456,825 A > C	C	0.3054	0.6552	SNV	2KB_upstream_variant, Upstream_transcript_variant	Likely‐pathogenic	20, 21
IL4	rs2243250	132,673,462 T > C	C	0.2042	0.4704	SNV	2KB_upstream_variant, Upstream_transcript_variant	Likely‐pathogenic	22
IL4	rs2070874	132,674,018 T > C	C	0.2073	0.667	SNV	5_prime_UTR_variant	Pathogenic	23, 24, 25, 26
IL6R	rs2228145	154,454,494 A > C	C	0.3874	0.4293	SNV	Missense_variant, Coding_sequence_variant, Intron_variant, Genic_downstream_transcript_variant	Benign, association	27, 28
IL9	rs1859430	135,894,824 G > A	A	0.0574	0.6509	SNV	Intron_variant	Pathogenic	29
IL10	rs1800871	206,773,289 A > G	G	0.3437	0.1446	SNV	2KB_upstream_variant, Upstream_transcript_variant	Pathogenic, Benign	10, 30, 31, 32
							Intron_variant		
							Genic_upstream_transcript_variant		
IL12A	rs2243115	159,988,493 T > G	G	0.06084	0.3955	SNV	2KB_upstream_variant, Upstream_transcript_variant, Intron_variant	Pathogenic	33, 34, 35
IL12A	rs6887695	159,395,637 G > C	C	0.4227	0.03432	SNV		Pathogenic, likely‐benign	36
IL22	rs1179251	68,251,271 C > G	G	0.3201	0.5175	SNV	Intron_variant	Pathogenic	37, 38, 39
TGFB1	rs1800469	41,354,391 A > G	G	0.4878	0.9249	SNV	2KB_upstream_variant, Upstream_transcript_variant, 500B_downstream_variant, Downstream_transcript_variant	Pathogenic, benign	40, 41
TBX21/ TBET	rs4794067	47,731,462 T > C	C	0.1203	0.3724	SNV	2KB_upstream_variant, Upstream_transcript_variant	Pathogenic, risk factor	42, 43
STAT1	rs3771300	190,970,870 T > G	G	0.2965	0.7363	SNV	Intron_variant, Genic_downstream_transcript_variant	Pathogenic	44
STAT3	rs744166	42,362,183 A > G	G	0.3352	1	SNV	Intron_variant, Genic_ upstream_transcript_variant	Pathogenic	45, 46
STAT5B	rs6503691	42,242,072 C > T	T	0.2219	0.4956	SNV	Intron_variant	Pathogenic	36, 47
STAT6	rs324011	57,108,399 C > T	T	0.2483	0.3124	SNV	Intron_variant, Genic_ upstream_transcript_variant	Pathogenic	8
GATA3	rs3824662	8,062,245 C > A	A	0.3426	0.9168	SNV	Intron_variant	Likely‐pathogenic, benign	48, 49
NFKBIA	rs2233406	35,405,593 G > A	A	0.09756	0.5985	SNV	2KB_upstream_variant, Upstream_transcript_variant	Pathogenic, benign	50
ITGAM	rs4597342	31,332,448 C > T	T	0.2411	0.519	SNV	3_prime_UTR_variant, Non_coding_transcript_variant	Likely‐pathogenic	51
							Genic_downstream_transcript_variant		
PTPN22	rs2488457	113,872,746 G > C	C	0.3633	0.9189	SNV	Intron_variant, 2KB_upstream_variant	Risk factor, likely‐benign, pathogenic	52
							Upstream_transcript_variant		
PARP1	rs1805414	226,385,663 G > A	A	0.229	0.7897	SNV	Synonymous_variant, Coding_sequence_variant	Likely‐pathogenic	53, 54
PARP1	rs907187	226,407,946 C > G	G	0.415	0.7701	SNV	5_prime_UTR_variant	Likely‐pathogenic	55, 56, 57

Abbreviations: HWE, Hardy–Weinberg equilibrium; MAF, minor allele frequency (>1%); SNP, single‐nucleotide polymorphisms.

### 3.2 Relationship between immune‐related genetic polymorphisms and lymphoma susceptibility

Five genetic models (dominant model, recessive model, overdominant model, codominant model and additive model) and allele model were used to analyze the relationship between immune‐related SNPs and lymphoma. After preliminary screening by χ^2^ test or Fisher's exact test, the influence of age or gender on the susceptibility of lymphoma was excluded. The results of Univariate binary logistic regression analysis showed that rs12979860 of IFNL3 was significantly associated with lymphoma susceptibility under dominant, additive and allele models, and patients with the T genotype were 2.049 times more susceptible to lymphoma than patients with C genotype (all *p* < 0.05). The rs1800629 of TNFA was also significantly associated with the susceptibility to lymphoma in the dominant, overdominant, codominant, additive and allelic models, and the risk of patients with genotype A was lower than that with genotype G (all *p* < 0.05), indicating the protective role for genotype A. For IL4, the rs2070874 and rs2243250 were correlated with the risk of lymphoma in the dominant, overdominant and codominant models, and the patients with CT genotype were significantly lower than those with the TT genotype. The OR of these two SNPs were 0.575 (0.386–0.857) and 0.568 (0.379–0.849), respectively, suggesting that the C genotype may be a protective factor. In addition, the risk of the AA genotype in rs1805414 of PARP1 was significantly lower than that of the GG genotype (OR = 0.375, 95% CI = 0.148–0.950, *p* = 0.043) under the codominant model.

As JAK–STAT and NF‐κB pathways were involved in lymphoma, we first found that the rs744166 of STAT3 and rs6503691 of STAT5B contributed to lymphoma susceptibility under codominant, recessive, codominant, and allele models (all *p* < 0.05). Secondly, our results showed that the rs2233406 of NFKBIA in NF‐κB pathway was significantly associated with the susceptibility of lymphoma in a variety of models (dominant, overdominant, codominant, additive, or allele models). And the mutation frequency of genotype A in lymphoma patients was significantly lower than that in controls, while genotype G was significantly higher in patients with lymphoma (*p* = 0.006), which indicated that genotype G is a risk factor for lymphoma susceptibility (Table 3). Therefore, our results reveal that some of our immune‐related genetic polymorphisms are associated with the lymphoma susceptibility.

**TABLE 3:** Association between selected SNPs and lymphoma susceptibility.

Gene	SNPs	Model	Genotype/allele	Controls, *n*	Cases, *n*	OR (95% CI)	Adjusted *p*‐value
IFNL3	rs12979860	Dominant	CC	195	205	2.014 (1.095–3.704)	0.036
			CT/TT	17	36		
		Additive	CC	195	205	2.041(1.132–3.679)	0.028
			CT	17	34		
			TT	0	2		
		Allele	C	407	444	2.049 (1.139–3.687)	0.028
			T	17	38		
TNFA	rs1800629	Dominant	GG	180	220	0.475 (0.257–0.877)	0.025
			GA/AA	31	18		
		Overdominant	GG/AA	182	220	0.513 (0.276–0.955)	0.050
			GA	29	18		
		Codominant	GG	180	220		
			GA	29	18	0.508 (0.273–0.944)	0.045
			AA	2	0	NA	0.981
		Additive	GG	180	220	0.468 (0.259–0.845)	0.017
			GA	29	18		
			AA	2	0		
		Allele	G	389	458	0.463 (0.257–0.836)	0.015
			A	33	18		
IL4	rs2070874	Dominant	TT	122	163	0.617 (0.420–0.907)	0.014
			CT/CC	91	75		
		Overdominant	TT/CC	131	175	0.575 (0.386–0.857)	0.007
			CT	82	63		
		Codominant	TT	122	163		
			CT	82	63	0.575(0.384–0.861)	0.007
			CC	9	12	0.998(0.408–2.444)	0.982
		Allele	T	326	389	0.729 (0.528–1.007)	0.056
			C	100	87		
IL4	rs2243250	Dominant	TT	122	164	0.610 (0.414–0.900)	0.013
			CT/CC	89	73		
		Overdominant	TT/CC	131	176	0.568 (0.379–0.849)	0.006
			CT	80	61		
		Codominant	TT	122	164		
			CT	80	61	0.567(0.377–0.852)	0.007
			CC	9	12	0.992(0.405–2.429)	0.978
		Allele	T	324	389	0.722 (0.522–1.001)	0.053
			C	98	85		
STAT3	rs744166	Codominant	AA	99	101		
			GA	97	104	1.051(0.710–1.555)	0.813
			GG	17	34	1.960(1.029–3.736)	0.037
		Recessive	AA/GA	196	205	1.383 (1.017–1.880)	0.035
			GG	17	34		
		Allele	A	295	306	1.266 (0.959–1.671)	0.092
			G	131	172		
STAT5B	rs6503691	Codominant	CC	138	139		
			CT	68	83	1.212(0.814–1.804)	0.366
			TT	7	18	2.553(1.034–6.306)	0.040
		Allele	C	344	361	1.383 (1.006–1.900)	0.047
			T	82	119		
NFKBIA	rs2233406	Dominant	GG	163	205	0.552 (0.340–0.895)	0.010
			AG/AA	49	34		
		Overdominant	GG/AA	167	206	0.594 (0.363–0.974)	0.025
			AG	45	33		
		Codominant	GG	163	205		
			AG	45	33	0.583(0.356–0.956)	0.020
			AA	4	1	0.199(0.022–1.796)	0.148
		Additive	GG	163	205	0.556 (0.355–0.872)	0.007
			AG	45	33		
			AA	4	1		
		Allele	G	371	443	0.553 (0.353–0.866)	0.006
			A	53	35		
PARP1	rs1805414	Codominant	GG	119	148		
			AG	79	84	0.855 (0.578–1.264)	0.469
			AA	15	7	0.375(0.148–0.950)	0.043
		Allele	G	317	380	0.750 (0.550–1.024)	0.082
			A	109	98		

Abbreviations: CI, confidence interval; OR, odds ratio; SNP, single nucleotide polymorphisms.

### 3.3 The immune‐related genetic polymorphisms are associated with the Ann Arbor stage in lymphoma

Patients with lymphoma have various degrees of lymph node or organ involvement, and the accurate and repeatable staging is crucial for the treatment and prognosis of lymphoma. In this study, according to the Ann Arbor Stage, this batch of lymphoma patients were divided into four Stages: I, II, III and IV.

For all lymphoma patients, the results of logistic regression analysis showed that patients with CA/CC genotype of IL6R rs2228145 had higher stage than patients with AA genotype under the dominant model (OR = 2.682, 95% CI = 1.084–7.033, *p* = 0.034), and patients with CA genotype had higher stage than patients with AA genotype under codominant model (OR = 2.741, 95% CI = 0.999–7.520, *p* = 0.048). In addition, the rs6503691 CT genotype of STAT5B was significantly different from that of the CC/TT genotype in different stages under the overdominant model (*p* = 0.030) (Table 5).

Due to the large scale of the MBN group, we further analyzed the relationship between the Ann Arbor stage and SNPs for the MBN lymphoma patients. Logistic regression analysis showed that the differences between the rs2228145 and the rs6503691 at different stages in MBN patients were similar to that in all lymphoma patients. Moreover, the rs6887695 genotype of IL12A was significantly different in four stages under codominant, recessive, additive and allele models (*p* < 0.05), and the risk of progression of lymphoma with C allele was much lower than that of the G allele (OR = 0.529, 95% CI = 0.280–1.001, *p* = 0.048), indicating that genotype G is a risk factor for lymphoma progression (Table 4).

**TABLE 4:** Association between Ann Arbor stage and SNPs in lymphoma.

Gene	SNPs	Model	Genotype/allele	Ann Arbor stage				OR (95% CI)	Adjusted *p*‐value
				I	II	III	IV		
MBN group and Non‐MBN group									
IL6R	rs2228145	Dominant	AA	13	8	30	45	2.682 (1.084–7.033)	0.034
			CA/CC	8	17	55	65		
		Codominant	AA	13	8	30	45		
			CA	6	13	41	51	2.741 (0.999–7.520)	0.048
			CC	2	4	14	14	2.506 (0.535–11.731)	0.235
		Allele	A	32	29	101	141	1.996 (0.989–4.375)	0.062
			C	10	21	69	79		
STAT5B	rs6503691	Overdominant	CC/TT	9	19	52	77	0.360 (0.141–0.889)	0.030
			CT	12	6	32	33		
		Allele	C	30	42	124	165	0.808 (0.409–1.692)	0.561
			T	12	8	44	55		
MBN group									
IL6R	rs2228145	Dominant	AA	12	5	28	41	3.000 (1.112–8.953)	0.043
			CA/CC	6	12	44	54		
		Codominant	AA	12	5	26	41		
			CA	4	8	31	41	3.333 (1.029–10.799)	0.050
			CC	2	4	11	13	2.333 (0.491–11.096)	0.331
		Allele	A	28	18	83	123	2.125 (0.983–5.118)	0.085
			C	8	16	53	67		
IL12A	rs6887695	Codominant	GG	4	5	30	31		
			GC	7	8	24	51	0.719 (0.202–2.560)	0.613
			CC	7	4	13	13	0.260 (0.071–0.955)	0.033
		Recessive	GG/GC	11	13	54	82	0.316 (0.115–0.920)	0.020
			CC	7	4	13	13		
		Additive	GG	4	5	30	31	0.492 (0.242–0.967)	0.034
			GC	7	8	24	51		
			CC	7	4	13	13		
		Allele	G	15	18	84	113	0.529 (0.280–1.001)	0.048
			C	21	16	50	77		
STAT5B	rs6503691	Overdominant	CC/TT	7	12	41	65	0.354 (0.125–0.944)	0.045
			CT	11	5	29	30		
		Allele	C	25	27	95	140	0.833 (0.404–1.823)	0.617
			T	11	7	39	50		

Abbreviations: CI, confidence interval; OR, odds ratio; SNP, single nucleotide polymorphisms.

### 3.4 Relationship between immune‐associated genetic polymorphisms and peripheral blood count and LDH

Clinical variables, especially the peripheral blood count and LDH, were reported to be involved in the lymphoma.^64^ We analyzed the relationship between immune‐related SNPs and peripheral blood count or LDH. In our study, a WBC higher than 15 × 10^9^/L was defined as a high WBC, no higher than 15 × 10^9^/L was defined as a low WBC,^65^ a PLT not higher than 250 × 10^9^/L is defined as a low PLT, PLT higher than 250 × 10^9^/L is a high PLT; HGB 110 g/L or lower is a low HGB, higher than 110 g/L is defined as a high HGB.^66^ In addition, according to the upper limit of the reference range of our hospital (≤ 230 U/L), we called abnormal increased LDH when it was >230 U/L. We performed binary logistic regression analysis in all lymphoma cases to determine the SNPs that may affect the clinical indicators, and to explore the roles of WBC, PLT, HGB or LDH in the prognosis of lymphoma.

The results of the binary logistic regression analysis showed that lymphoma patients with GA genotype in rs744166 of STAT3 were more likely to have low PLT than AA or AA/GG under the overdominant and codominant models (OR = 1.873, 95% CI = 1.060–3.307, *p* = 0.032; OR = 1.827, 95% CI = 1.071–3.117, *p* = 0.028) (Table 5). As for HGB, the genotype frequency of rs3824662 in GATA3 under dominant, overdominant or codominant models was significantly different between high and low HGB groups. Patients with CA or CA/AA genotype were more likely to develop anemia than those with CC genotype (OR = 2.019, 95% CI = 1.105–3.690, *p* = 0.021; OR = 1.867, 95% CI = 1.048–3.326, *p* = 0.031), indicating that A allele may be a negative factor. And the patients with rs907187 carrying CG genotype of PARP1 was 2.091 and 2.209 times higher than that in patients with CC or CC/GG (OR = 2.091, 95% CI = 1.092–4.005, *p* = 0.030; OR = 2.209, 95% CI = 1.239–3.940, *p* = 0.009) (Table 5). However, we cannot observe a significant relationship between SNPs and WBC count.

**TABLE 5:** Association between peripheral blood counts and SNPs in lymphoma.

Gene	SNPs	Model	Genotype/allele	Peripheral Blood Counts		OR (95% CI)	Adjusted *p*‐value
				PLT > 250, *n*	PLT ≤ 250,*n*		
STAT3	rs744166	Overdominant	AA/GG	62	73	1.827(1.071–3.117)	0.028
			GA	33	71		
		Codominant	AA	47	54		
			GA	33	71	1.873 (1.060–3.307)	0.032
			GG	15	19	1.102 (0.504–2.409)	0.814
		Allele	A	127	179	1.228(0.836–1.803)	0.300
			G	63	109		

				HGB > 110, *n*	HGB ≤ 110,*n*		
GATA3	rs3824662	Dominant	CC	82	24	1.867 (1.048–3.326)	0.031
			CA/AA	86	47		
		Overdominant	CC/AA	102	32	1.884 (1.075–3.300)	0.027
			CA	66	39		
		Codominant	CC	82	24		
			CA	66	39	2.019 (1.105–3.690)	0.021
			AA	20	8	1.367 (0.535–3.490)	0.470
		Allele	C	230	87	1.372 (0.912–2.064)	0.129
			A	106	55		
PARP1	rs907187	Overdominant	CC/GG	88	24	2.209 (1.239–3.940)	0.009
			CG	78	47		
		Codominant	CC	59	17		
			CG	78	47	2.091 (1.092–4.005)	0.030
			GG	29	7	0.838 (0.313–2.246)	0.728
		Allele	C	196	81	1.085 (0.729–1.616)	0.701
			G	136	61		

Abbreviations: CI, confidence interval; OR, odds ratio; SNP, single nucleotide polymorphisms.

As for the importance of LDH in lymphoma,^67^ we further analyzed the relationship between the genetic polymorphism and LDH. Our results showed that the rs2069762 of IL2 had a significant difference between the high and low groups of LDH used by overdominant, codominant and recessive models, and the risk of abnormal increased LDH in patients with CA genotype was 2.021 or 2.289 times higher than that in patients with AA or AA/CC genotype, respectively (*p* < 0.05). Under the codominant and recessive model, the risk of LDH abnormality in patients with rs1800871 GG genotype of IL10 was 3.603 times higher than that in patients with AA genotype. In the codominant, additive or allele models, there was a significant difference of rs3824662 genotype of GATA3 between the two LDH groups, and the patients with AA genotype had a lower risk of increased LDH than those with CC genotype (OR = 0.392, 95% CI = 0.160–0.965, *p* = 0.040) (Table 6).

**TABLE 6:** Association between LDH count and SNPs in lymphoma.

Gene	SNPs	Model	Genotype/allele	LDH ≤230,*n*	LDH > 230, *n*	OR (95% CI)	Adjusted *p*‐value
IL2	rs2069762	Overdominant	AA/CC	72	58	2.289 (1.318–3.975)	0.003
			CA	32	59		
		Codominant	AA	57	52		
			CA	32	59	2.021 (1.141–3.579)	0.015
			CC	15	6	0.438 (0.158–1.214)	0.110
		Recessive	AA/CA	89	111	0.566 (0.346–0.928)	0.023
			CC	15	6		
		Allele	A	146	163	1.026(0.683–1.542)	0.905
			C	62	71		
IL10	rs1800871	Codominant	AA	49	51		
			GA	56	53	0.909 (0.528–1.565)	0.720
			GG	4	15	3.603 (1.118–11.615)	0.034
		Recessive	AA/GA	105	104	1.946 (1.103–3.433)	0.023
			GG	4	15		
		Allele	A	154	155	1.289(0.868–1.913)	0.223
			G	64	83		
GATA3	rs3824662	Codominant	CC	43	58		
			CA	50	52	0.771 (0.443–1.341)	0.375
			AA	17	9	0.392 (0.160–0.965)	0.040
		Additive	CC	43	58	0.673 (0.454–0.998)	0.049
			CA	50	52		
			AA	17	9		
		Allele	C	136	168	0.675(0.457–0.996)	0.048
			A	84	70		

Abbreviations: CI, confidence interval; OR, odds ratio; SNP, single nucleotide polymorphisms.

### 3.5 The immune‐related genetic polymorphisms are associated with the survival of lymphoma patients

**FIGURE 1:**
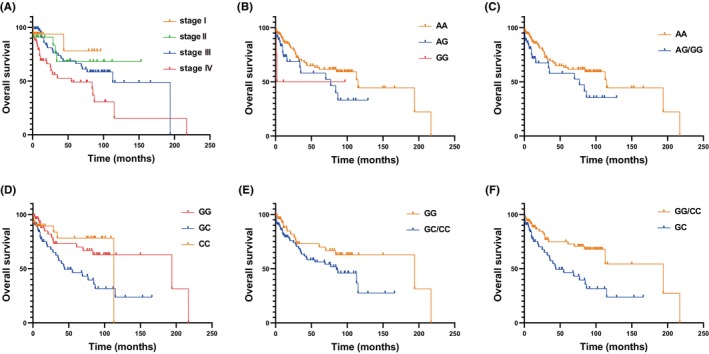
The OS of Ann Arbor stages and two genetic polymorphisms in lymphoma patients. (A). The OS of Ann Arbor Stage I, II, III, IV. (B).The OS of lymphoma patients with AA, AG, GG genotypes in rs2069718 under codominant and additive models. (C). The OS of lymphoma patients with AA, AG/GG genotypes in rs2069718 under dominant model. (D). The OS of lymphoma patients with CC, GC, GG genotypes in rs6887695 under codominant model. (E). The OS of lymphoma patients with GG, GC/CC genotypes in rs6887695 under dominant model (F). The OS of lymphoma patients with GG/CC, GC genotypes in rs6887695 under overdominant model.

First, we investigated the relationship of OS with clinical characteristics. We found that there existed significant differences between the age ≥ 60 group and the age < 60 group (*p* = 0.044). And there was no significant difference in OS between males and females (*p* > 0.05). In addition, OS among the four stages of Ann Arbor stage exhibited remarkable differences (*p* = 0.0037), among which the OS was negatively related to the clinical stages (Figure 1A).

We further analyzed the relationship between SNPs and the OS of lymphoma patients under five genetic models and allele models. Univariate Cox proportional hazards regression model showed that the frequency of rs2069718 genotype of IFNG was significantly associated with the survival of lymphoma patients under dominant, codominant, additive and allele models (all *p* < 0.05) (Figure 1B,C). The risk about survival of lymphoma patients with the AG genotype was 1.760 times higher than that of the AA genotype, and the G allele genotype was 1.726 times higher than that of the A allele, suggesting that the G genotype was a poor prognostic factor. Consistently, the result of Log‐rank (Mantel‐Cox) analysis showed that the survival time of AG/GG genotype was significantly shorter than that of AA genotype (*p* = 0.032). In addition, the rs6887695 of IL12A was also significantly associated with the survival of lymphoma under the dominant, overdominant or codominant models. In codominant or overdominant models, the risk about survival of patients with GC genotype was 2.3 times higher than that of GG or GG/CC genotype (2.301 (1.254–4.225) or 2.380 (1.391–4.072)), and the survival time was shorter than that of GG or GG/CC (Figure 1D,F). After Bonferroni multiple corrections, the rs6887695 was also significantly associated with OS under the overdominant model. Besides, in the dominant model, patients with GC/CC genotype had an increased risk of OS compared with that genotype GG (HR = 1.825, 95% CI = 1.011–3.294) in rs6887695 of IL12A (Figure 1E). Therefore, genotype GC may be an adverse prognostic factor (Table 7).

**TABLE 7:** Association between OS and SNPs in lymphoma.

Gene	SNPs	Model	Genotype/allele	Survival	Death	HR (95% CI)	Adjusted *p*‐value
IFNG	rs2069718	Dominant	AA	120	39	1.823 (1.047–3.174)	0.030
			AG/GG	31	19		
		Codominant	AA	120	39		
			AG	29	17	1.760 (0.990–3.130)	0.049
			GG	2	2	2.621(0.630–10.905)	0.153
		Additive	AA	120	39	1.703 (1.066–2.722)	0.020
			AG	29	17		
			GG	2	2		
		Allele	A	269	95	1.726 (1.074–2.776)	0.020
			G	33	21		
IL12A	rs6887695	Dominant	GG	59	17	1.825 (1.011–3.294)	0.040
			GC/CC	92	42		
		Overdominant	GG/CC	90	24	2.380 (1.391–4.072)	0.001
			GC	61	35		
		Codominant	GG	59	17		
			GC	61	35	2.301 (1.254–4.225)	0.006
			CC	31	7	0.903 (0.367–2.217)	0.872
		Allele	G	179	69	1.113 (0.768–1.614)	0.535
			C	123	49		

Abbreviations: CI, confidence interval; HR, Hazard ratio; SNP, single nucleotide polymorphisms.

**FIGURE 2:**
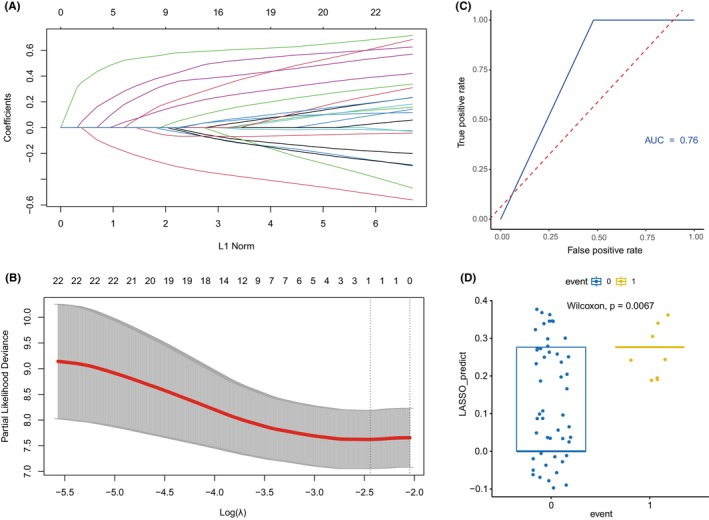
The selection of the variables by LASSO regression under overdominant model. (A). The variation characteristics of the coefficient of variables. (B). The selection process of the optimum value of the parameter *λ* in the LASSO regression model by ten‐fold cross‐validation method. (C). ROC curve of overdominant model by LASSO regression. (D). The wilcoxon analyis between two survival states in test subsets under overdominant model.

We performed the LASSO regression to further validate our analysis results. LASSO regression and univariate Cox regression indicated that the rs2069718 of IFNG and rs6887695 of IL12A were correlated with the survival of the lymphoma patients under dominant and codominant models (all *p* < 0.05), and the AUC (Area Under Curve) were all greater than 0.62. The rs2069718 of IFNG was associated with the patients survival under additive model, and the rs6887695 of IL12A was associated with the patients' survival under the overdominant model, and the AUC of two SNPs were 0.69 (HR = 2.876, 95% CI = 1.488–5.559, *p* = 0.002) and 0.76 (HR = 2.246, 95% CI = 1.230–4.101, *p* = 0.008) separately. However, we cannot obtain some meaningful markers under the recessive model. The LASSO regression of the overdominant model was shown in Figure 2 and other genetic models can be seen in (Figure S1: codominant model; Figure S2: additive model; Figure S3: dominant model). All the results of five genotype models we got by LASSO regression models were generally consistent with the results obtained by the Cox proportional hazards models. The LASSO does not focus on subsets but rather defines a continuous shrinking operation that can produce coefficients that are exactly zero. Therefore, we identified that the SNPs associated with lymphoma patients' survival were the rs2069718 of IFNG and the rs6887695 of IL12A.

Finally, combined with the age, gender, Ann Arbor stage and whole blood count, the Cox proportional hazards model and LASSO regression model with SNPs were used for multivariate analysis. After age and gender adjusted, it was found that Ann Arbor stage (OR = 1.764, 95% CI = 1.227–2.535) and HGB (OR = 2.613, 95% CI = 1.527–4.473) and LDH (OR = 2.087, 95% CI = 1.194–3.647) have independent effects on OS. And HGB still had a significant effect on survival after stepwise regression (OR = 2.214, 95% CI = 1.216–4.030, *p* = 0.009). By analyzing the two SNPs associated with HGB, we found that the CA/AA genotype of rs3824662 had poorer OS than CC genotype, and the CA genotype had shorter OS than CC/AA or CC genotype. And the CG genotype in rs907187 had shorter OS than CC/GG or CC genotype, which corresponded to the high risk of anemia of the CG genotype, indicating that the G genotype was a poor prognostic factor.

### 3.6 Association of the genetic polymorphisms and mRNA expression levels of IFNG and IL12A

The results of statistical analysis in our research found that rs2069718 of IFNG and rs6887695 of IL12A were significantly associated with OS in lymphoma patients. Therefore, in order to further determine the role of the two genetic polymorphisms on the progression and prognosis of lymphoma, we explored the association of the two genetic polymorphisms and the mRNA expression levels of IFNG and IL12A in some available lymphoma samples.

**FIGURE 3:**
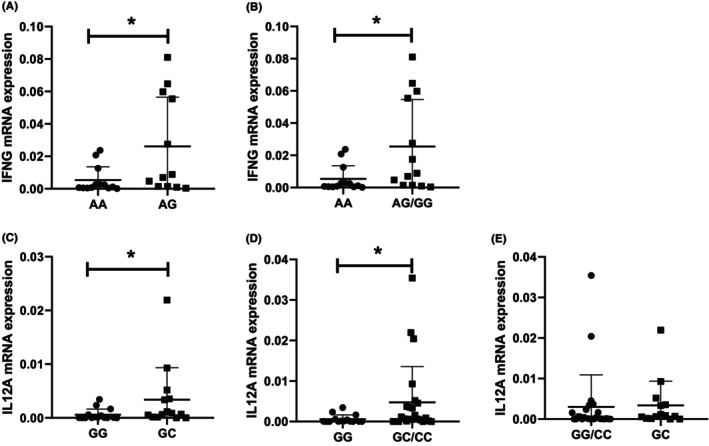
The mRNA expression of IFNG and IL12A. (A). Expression of IFNG mRNA in lymphoma patients with the AA and AG genotypes under codominant model. (B). Expression of IFNG mRNA in lymphoma patients with the AA and AG/GG genotypes under dominant model. (C). Expression of IL12A mRNA in lymphoma patients with the GG and GC genotypes under codominant model. (D). Expression of IL12A mRNA in lymphoma patients with the GG and GC/CC genotypes under dominant model. (E). Expression of IL12A mRNA in lymphoma patients with the GG/CC and GC genotypes under overdominant model. *, *p* < 0.05.

For IFNG, we analyzed the expressions in 26 lymphoma patients, which included the rs2069718 polymorphisms of 13 AA, 12 AG, and 1 GG. Under the codominant model, patients with AG genotype had a higher mRNA expression of IFNG than AA genotype (*p* = 0.0383, Figure 3A). And in the dominant model, the expression level of IFNG in lymphoma patients with AG/GG genotype was also higher than those with AA genotype (*p* = 0.0284, Figure 3B). These results were consistent with the results of rs2069718 about OS. Among the 40 patients participated in the analysis of mRNA expression of IL12A, 15 patients with the GG genotype, 15 with GC genotype and 10 with CC genotype. Under the codominant and dominant model, GC genotype and GC/CC genotype had much high mRNA expression level of IL12A than GG genotype (all *p* < 0.05) (Figure 3C,D). However, there was no significant difference of IL12A mRNA expression between GG/CC and GC genotypes under overdominant model (Figure 3E).

## 4 DISCUSSION

In this study, we performed genetic polymorphisms analysis of 24 immune‐related genes based on 245 lymphoma patients and 213 healthy controls to identify immune‐related SNPs that may be related to the susceptibility, progression, and prognosis of lymphoma. After strictly statistical analysis, we found that 15 immune‐related SNPs were associated with lymphoma susceptibility and clinical characteristics, including Ann Arbor stage, peripheral blood counts, LDH and OS. Especially, rs6887695 was closely related to the survival of lymphoma patients after bonferroni multiple correction.

Previous studies have demonstrated that most genetic polymorphisms were significantly associated with lymphoma susceptibility. For example, IL‐10 polymorphisms were reported to be associated with the risk of lymphoma,^10^ and rs1800871‐CC may play a pathogenic role in the occurrence of non‐Hodgkin's lymphoma.^31^ Besides, the TG/GG of rs2069762 in IL‐2 increased the risk of non‐Hodgkin's lymphoma by more than three times, and was more likely to develop adverse outcomes.^20^ In our results, we found that a total of eight SNPs contributed to lymphoma susceptibility under at least one genetic model, which were significantly different between lymphoma patients and healthy controls. These SNPs include cytokines (IFNL3, TNFA, IL4), genes of JAK–STAT and NF‐κB pathways (STAT3, STAT5B, NFKBIA), and PARP1 which plays an important role in DNA repair and apoptosis. A study on myeloproliferative neoplasms (MPN) revealed that the CC genotype of IFNL3 rs12979860 was significantly associated with hematologic response to interferon‐α treatment, with a significantly higher complete response rate compared with non‐CC.^68^ Besides, Annibali et al. found that the T allele of rs12979860 in IFNL3 significantly increased the risk of cytomegalovirus infection in patients with hematological malignancies undergoing autologous stem cell transplantation.^19^ And we also found that the T allele in rs12979860 increased the risk of lymphoma susceptibility compared with the C allele, indicating the adverse effect of T in lymphoma.

In addition, in the JAK–STAT pathway, we found the GG genotype of STAT3 rs744166 compared with AA and the T allele of STAT5B rs6503691 compared with C were significantly associated with lymphoma susceptibility. Consistent with our results, other study reported that the GG genotype of rs744166 correlated with immunotherapy failure in aplastic anemia patients^46^ and rs6503691 TT/CT genotypes were closely associated with recurrent genetic abnormalities in acute myeloid leukemia (AML) patients.^36^ For genes in NF‐κB pathway, we found that lymphoma susceptibility of the A allele was significantly decreased in rs2233406 of NFKBIA. And Zhang et al. found that another genetic polymorphism in NFKBIA, rs696, also exhibited significantly decreased susceptibility in Hodgkin lymphoma,^50^ suggesting the important role of NFKBIA genetic polymorphism in lymphoma.

The immune‐related genetic polymorphisms have been reported to be related to many clinical characteristics that are involved in poor prognosis, such as lower HGB levels, higher WBC, lower PLT counts, higher LDH levels, higher bone marrow involvement, and advanced diseases.^20^ We integrated and investigated the clinical characteristics of lymphoma patients, including peripheral blood counts, LDH counts, Ann Arbor stages, and OS. Rigorous statistical analysis found that a total of nine SNPs was significantly associated with clinical indices. The rs1800871 of IL10 showed significant difference in LDH level, and the CC genotype suggested a higher risk of abnormally increased LDH than the TT genotype. Consistently, Liu et al. also found that patients with TT genotype in rs1800871 had higher complete remission (CR) or overall response rate, and longer progression‐free survival (PFS).^32^ Kim et al. also showed that rs1800871 had a significant correlation with the prognosis of NHL.^30^ Moreover, rs2228145, which is closely associated with Ann Arbor stage, has been reported to affect the balance of the IL‐6 activities between pro‐inflammatory and anti‐inflammatory. In patients homozygous for the major alleles of rs2228145, serum CRP levels increased before transplantation, which suggested the major allele of rs2228145 was an adverse factor.^27^ In our study, patients with the major allele AA had much lower OS than CC genotype, while in the analysis of Ann Arbor stage, AA genotype has lower stage than CA/CC genotype, which needs to be studied in the future.

For rs3824662 of GATA3 associated with HGB, we found that the CA genotype was more likely to develop a lower HGB than CC genotype. Consistently, it was reported that the presence of A allele of rs3824662 correlated with an increased risk of relapse and adverse outcomes in acute lymphoblastic leukemia patients.^49^ The mortality rate in AA genotype carriers was significantly higher than that in CA + CC carriers.^48^ That may be due to the rs3824662 located in the GATA3 enhancer region, which affected the gene expression and functions of GATA3. Similarly, we found that patients who carried the CG genotype contributed to a lower HGB than CC genotype in the PARP1 rs907187 which was located in the promoter region. A recent study showed that the SNPs of PARP1 significantly altered the response of cancer cells to treatment, due to affecting the expression of PARP1.^55^ However, Avitabile M showed that the G allele of rs907187 was associated with a better response to induction therapy in neuroblastoma patients, supporting the potential role of PARP1 as a candidate gene for neuroblastoma treatment failure.^56^ The opposite result may be due to the heterogeneity of different tumors.

IFN is considered to have antiviral and immunomodulatory effects in vivo, which triggers JAK–STAT pathway, and ultimately up‐regulates the expression of interferon stimulating genes.^69^, ^70^ Our results showed that IFNG rs2069718 was associated with the survival of lymphoma patients under dominant model, and the G allele of rs2069718 had a lower OS. Similarly, other study reported that G allele of rs2069718 contributed to the susceptibility and severity of mixed connective tissue disease (MCTD).^71^ IL‐12 plays a central role in the cellular and humoral pathways, which is mainly produced by antigen‐presenting cells and acts on T cells and natural killer (NK) by inducing IFN‐γ production.^72^, ^73^ Recently, the IPI‐based immune prognostic model based on eight genes including IL12A had independent prognostic significance in DLBCL patients, which provided an immunological perspective for elucidating the tumor progression mechanism of DLBCL, and were also useful for DLBCL immunotherapy.^74^ We found that the GC genotype had the lowest OS, and the GG and CC genotypes had similar OS levels in lymphoma patients.

In summary, we found some novel molecular markers related to lymphoma susceptibility and disease progression. Especially, IFNG rs2069718 and IL‐12A rs6887695 were significantly associated with OS, and the mRNA expressions of the two genes were also associated with OS of lymphoma patients. We believe that it can be used as a preliminary screen for gene polymorphisms studies and has important exploration value for the prognosis of lymphoma. However, there are also some limitations in our research. First, our patients were recruited all from one hospital which may be regionally biased due to the specific populations, so we may need a multicenter meta‐analysis in the future. Second, our SNPs analysis did not cover all genes related to the immune regulatory pathways, which may result in insufficient or uncomprehensive results. Third, our findings are based on the normal peripheral blood and LDH counts of all healthy controls, which may be affected by the lack of the clinical parameters of healthy controls. Furthermore, due to the limited scale and the heterogeneity of the lymphoma patients, our results may need to be further confirmed in the future.

## AUTHOR CONTRIBUTIONS

**Chaoyang Gu:** Formal analysis (equal); writing – original draft (equal). **Can Can:** Funding acquisition (equal). **Jinting Liu:** Conceptualization (equal). **Yihong Wei:** Data curation (equal). **Xinyu Yang:** Investigation (equal). **Xiaodong Guo:** Conceptualization (equal); data curation (equal). **Ruiqing Wang:** Methodology (equal). **Wenbo Jia:** Data curation (equal). **Wancheng Liu:** Data curation (equal). **Daoxin Ma:** Resources (equal); writing – review and editing (equal).

## CONFLICT OF INTEREST STATEMENT

The authors declare no conflict of interest.

## ETHICS STATEMENT

The studies involving humans were conducted in accordance with the Declaration of Helsinki, and approved by the Ethics Committee on Scientific Research of Shandong University Qilu hospital (KYLL‐202204‐059).

## Supporting information


Figure S1.

Figure S2.

Figure S3.

## Data Availability

The data that support the findings of this study are available from the corresponding author upon reasonable request.

## References

[1] Zhao X , Zhang C , Hua M , et al. NLRP3 inflammasome activation plays a carcinogenic role through effector cytokine IL‐18 in lymphoma. Oncotarget. 2017;8:108571‐108583. 29312552 10.18632/oncotarget.21010 PMC5752465

[2] Autio M , Leivonen SK , Brück O , et al. Immune cell constitution in the tumor microenvironment predicts the outcome in diffuse large B‐cell lymphoma. Haematologica. 2021;106:718‐729. 32079690 10.3324/haematol.2019.243626 PMC7927991

[3] Sheng L , Fu D , Cao Y , et al. Integrated genomic and transcriptomic analyses of diffuse large B‐cell lymphoma with multiple abnormal immunologic markers. Front Oncol. 2022;12:790720. 35237512 10.3389/fonc.2022.790720 PMC8882913

[4] Tun AM , Ansell SM . Immunotherapy in Hodgkin and non‐Hodgkin lymphoma: innate, adaptive and targeted immunological strategies. Cancer Treat Rev. 2020;88:102042. 32521386 10.1016/j.ctrv.2020.102042

[5] Lu T , Yu S , Liu Y , et al. Aberrant circulating Th17 cells in patients with B‐cell non‐Hodgkin's lymphoma. PLoS One. 2016;11:e0148044. 26812681 10.1371/journal.pone.0148044 PMC4727938

[6] Abramson JS . Anti‐CD19 CAR T‐cell therapy for B‐cell non‐Hodgkin lymphoma. Transfus Med Rev. 2020;34:29‐33. 31677848 10.1016/j.tmrv.2019.08.003

[7] Miao Y , Medeiros LJ , Li Y , et al. Genetic alterations and their clinical implications in DLBCL. Nat Rev Clin Oncol. 2019;16:634‐652. 31127191 10.1038/s41571-019-0225-1

[8] Butterbach K , Beckmann L , DE Sanjose S , et al. Association of JAK‐STAT pathway related genes with lymphoma risk: results of a European case‐control study (EpiLymph). Br J Haematol. 2011;153:318‐333. 21418178 10.1111/j.1365-2141.2011.08632.x

[9] Wang SS , Purdue MP , Cerhan JR , et al. Common gene variants in the tumor necrosis factor (TNF) and TNF receptor superfamilies and NF‐kB transcription factors and non‐Hodgkin lymphoma risk. PLoS One. 2009;4:e5360. 19390683 10.1371/journal.pone.0005360 PMC2669130

[10] Dai ZM , He AL , Zhang WG , et al. Association of the four common polymorphisms in interleukin‐10 (rs1800890, rs1800896, rs1800871, and rs1800872) with non‐Hodgkin's lymphoma risk: a meta‐analysis. Int J Clin Exp Med. 2014;7:4720‐4733. 25663969 PMC4307416

[11] Gaiolla RD , Moraes MPT , DE Oliveira DE . SNPs in genes encoding for IL‐10, TNF‐α, and NFκB p105/p50 are associated with clinical prognostic factors for patients with Hodgkin lymphoma. PLoS One. 2021;16:e0248259. 33684151 10.1371/journal.pone.0248259 PMC7939322

[12] Spalinger MR , Lang S , Gottier C , et al. PTPN22 regulates NLRP3‐mediated IL1B secretion in an autophagy‐dependent manner. Autophagy. 2017;13:1590‐1601. 28786745 10.1080/15548627.2017.1341453 PMC5612532

[13] Wan TWR , Smyth DJ , Merriman ME , et al. The PTPN22 locus and rheumatoid arthritis: no evidence for an effect on risk independent of Arg620Trp. PLoS One. 2010;5:e13544. 20975833 10.1371/journal.pone.0013544 PMC2958827

[14] Pan T , He Y , Chen H , et al. Identification and validation of a prognostic gene signature for diffuse large B‐cell lymphoma based on tumor microenvironment‐related genes. FRONT Oncol. 2021;11:614211. 33692952 10.3389/fonc.2021.614211 PMC7938316

[15] Mahe E , Akhter A , Le A , et al. PARP1 expression in mantle cell lymphoma: the utility of PARP1 immunohistochemistry and its relationship with markers of DNA damage. Hematol Oncol. 2015;33:159‐165. 10.1002/hon.2160 25143154

[16] Wang SS , Cerhan JR , Hartge P , et al. Common genetic variants in proinflammatory and other immunoregulatory genes and risk for non‐Hodgkin lymphoma. Cancer Res. 2006;66:9771‐9780. 17018637 10.1158/0008-5472.CAN-06-0324

[17] Wang SS , Vajdic CM , Linet MS , et al. Associations of non‐Hodgkin lymphoma (NHL) risk with autoimmune conditions according to putative NHL loci. Am J Epidemiol. 2015;181:406‐421. 25713336 10.1093/aje/kwu290 PMC4402340

[18] NUNEZ‐Marrero A , Arroyo N , Godoy L , et al. SNPs in the interleukin‐12 signaling pathway are associated with breast cancer risk in Puerto Rican women. Oncotarget. 2020;11:3420‐3431. 32973967 10.18632/oncotarget.27707 PMC7500104

[19] Annibali O , Piccioni L , Tomarchio V , et al. Impact of IFN lambda 3/4 single nucleotide polymorphisms on the cytomegalovirus reactivation in autologous stem cell transplant patients. PLoS One. 2018;13:e0200221. 30036376 10.1371/journal.pone.0200221 PMC6056038

[20] Mousa SM , Makhlouf MM , Mohammed ET , et al. The influence of interleukin‐2 gene polymorphisms on the risk and clinical outcome of non‐Hodgkin lymphoma. Indian J Hematol Blood Transfus. 2021;37:549‐554. 34744338 10.1007/s12288-020-01388-4 PMC8523617

[21] Song N , Han S , Lee KM , et al. Genetic variants in interleukin‐2 and risk of lymphoma among children in Korea. Asian Pac J Cancer Prev. 2012;13:621‐623. 22524835 10.7314/apjcp.2012.13.2.621

[22] Schoof N , VON Bonin F , Zeynalova S , et al. Favorable impact of the interleukin‐4 receptor allelic variant I75 on the survival of diffuse large B‐cell lymphoma patients demonstrated in a large prospective clinical trial. Ann Oncol. 2009;20:1548‐1554. 19515749 10.1093/annonc/mdp110

[23] Cho YA , Kim J . Association of IL4, IL13, and IL4R polymorphisms with gastrointestinal cancer risk: a meta‐analysis. J Epidemiol. 2017;27:215‐220. 28142034 10.1016/j.je.2016.06.002 PMC5394226

[24] Chu CN , Wang YC , Chang WS , et al. Association of interleukin‐4 polymorphisms with breast cancer in Taiwan. In Vivo. 2020;34:1111‐1116. 32354899 10.21873/invivo.11882 PMC7279797

[25] Jia Y , Xie X , Shi X , et al. Associations of common IL‐4 gene polymorphisms with cancer risk: a meta‐analysis. Mol Med Rep. 2017;16:1927‐1945. 28656227 10.3892/mmr.2017.6822 PMC5561993

[26] Chang WS , Wang SC , Chuang CL , et al. Contribution of interleukin‐4 genotypes to lung cancer risk in Taiwan. Anticancer Res. 2015;35:6297‐6301. 26504066

[27] Tvedt THA , Hovland R , Tsykunova G , et al. A pilot study of single nucleotide polymorphisms in the interleukin‐6 receptor and their effects on pre‐ and post‐transplant serum mediator level and outcome after allogeneic stem cell transplantation. Clin Exp Immunol. 2018;193:130‐141. 29513361 10.1111/cei.13124 PMC6037994

[28] Zhang JZ , Liu CM , Peng HP , et al. Association of genetic variations in IL‐6/IL‐6R pathway genes with gastric cancer risk in a Chinese population. Gene. 2017;623:1‐4. 28442395 10.1016/j.gene.2017.04.038

[29] Pasvenskaite A , Liutkeviciene R , Gedvilaite G , et al. The role of IL‐9 polymorphisms and serum IL‐9 levels in carcinogenesis and survival rate for laryngeal squamous cell carcinoma. Cell. 2021;10:601. 10.3390/cells10030601 PMC8001846 33803218

[30] Kim MK , Yoon KA , Park EY , et al. Interleukin‐10 polymorphisms in association with prognosis in patients with B‐cell lymphoma treated by R‐CHOP. Genomics Inform. 2016;14:205‐210. 28154512 10.5808/GI.2016.14.4.205 PMC5287125

[31] Lim YY , Chin YM , Tai MC , et al. Analysis of interleukin‐10 promoter single nucleotide polymorphisms and risk of non‐Hodgkin lymphoma in a Malaysian population. Leuk Lymphoma. 2015;56:163‐168. 24684230 10.3109/10428194.2014.907895

[32] Liu D , Wang Y , Dong M , et al. Polymorphisms in cytokine genes as prognostic markers in diffuse large B cell lymphoma patients treated with (R)‐CHOP. Ann Hematol. 2017;96:227‐235. 27822610 10.1007/s00277-016-2857-x

[33] Li CH , Shih LC , Hsu CL , et al. The contribution of interleukin‐12A genotypes to oral cancer risk in Taiwanese. Anticancer Res. 2020;40:3707‐3712. 32620609 10.21873/anticanres.14359

[34] Shi X , Jia Y , Xie X , et al. Single‐nucleotide polymorphisms of the IL‐12 gene lead to a higher cancer risk: a meta‐analysis based on 22,670 subjects. Genes Genet Syst. 2018;92:173‐187. 28408727 10.1266/ggs.16-00024

[35] Zheng Y , Wang M , Tian T , et al. Role of interleukin‐12 gene polymorphisms in the onset risk of cancer: a meta‐analysis. Oncotarget. 2017;8:29795‐29807. 28415696 10.18632/oncotarget.16080 PMC5444704

[36] Liu Q , Hua M , Yan S , et al. Immunorelated gene polymorphisms associated with acute myeloid leukemia. Clin Exp Immunol. 2020;201:266‐278. 32349161 10.1111/cei.13446 PMC7419888

[37] Wang H , Huang C , Liu Y , et al. Lack of association between interleukin‐22 gene polymorphisms and cancer risk: a case‐control study and a meta‐analysis. Int J Clin Oncol. 2020;25:521‐530. 31832882 10.1007/s10147-019-01595-8

[38] Wang YM , Li ZX , Tang FB , et al. Association of genetic polymorphisms of interleukins with gastric cancer and precancerous gastric lesions in a high‐risk Chinese population. Tumour Biol. 2016;37:2233‐2242. 26358252 10.1007/s13277-015-4022-x

[39] Zhang J , Zhao T , Xu C , et al. Four polymorphisms in the IL‐22 gene and the risk of cancer: a meta‐analysis. J Evid Based Med. 2018;11:101‐104. 29761647 10.1111/jebm.12296

[40] Chen G , Hu C , Lai P , et al. Association between TGF‐beta1 rs1982073/rs1800469 polymorphism and lung cancer susceptibility: an updated meta‐analysis involving 7698 cases and controls. Medicine (Baltimore). 2019;98:e18028. 31764821 10.1097/MD.0000000000018028 PMC6882652

[41] Das AP , Chopra M , Agarwal SM . Prioritization and meta‐analysis of regulatory SNPs identified IL6, TGFB1, TLR9 and MMP7 as significantly associated with cervical cancer. Cytokine. 2022;157:155954. 35810505 10.1016/j.cyto.2022.155954

[42] Thude H , Tiede P , Sterneck M , et al. Impact of TBX21, GATA3, and FOXP3 gene polymorphisms on acute cellular rejection after liver transplantation. HLA. 2019;93:97‐101. 30614205 10.1111/tan.13458

[43] Wang HL , Wang H , Wu Y , et al. Association of the rs17250932, rs4794067, and rs2240017 polymorphism in the TBX21 gene with autoimmune diseases: a meta‐analysis. Allergol Immunopathol (Madr). 2021;49:83‐90. 33938192 10.15586/aei.v49i3.80

[44] Zhu ZZ , Di JZ , Gu WY , et al. Association of genetic polymorphisms in STAT1 gene with increased risk of hepatocellular carcinoma. Oncology. 2010;78:382‐388. 20798561 10.1159/000320521

[45] Yan R , Lin F , Hu C , et al. Association between STAT3 polymorphisms and cancer risk: a meta‐analysis. Mol Genet Genomics. 2015;290:2261‐2270. 26063618 10.1007/s00438-015-1074-y

[46] Zhao L , Zhu H , Han B , et al. Influence of genetic polymorphisms of IL23R, STAT3, IL12B, and STAT4 on the risk of aplastic anemia and the effect of immunosuppressive therapy. Ann Hematol. 2018;97:685‐695. 29330562 10.1007/s00277-018-3227-7

[47] Kreil S , Waghorn K , Ernst T , et al. A polymorphism associated with STAT3 expression and response of chronic myeloid leukemia to interferon alpha. Haematologica. 2010;95:148‐152. 20065083 10.3324/haematol.2009.011510 PMC2805737

[48] Madzio J , Pastorczak A , Sedek L , et al. GATA3 germline variant is associated with CRLF2 expression and predicts outcome in pediatric B‐cell precursor acute lymphoblastic leukemia. Genes Chromosomes Cancer. 2019;58:619‐626. 30859636 10.1002/gcc.22748

[49] PEREZ‐Andreu V , Roberts KG , Harvey RC , et al. Inherited GATA3 variants are associated with Ph‐like childhood acute lymphoblastic leukemia and risk of relapse. Nat Genet. 2013;45:1494‐1498. 24141364 10.1038/ng.2803 PMC4039076

[50] Zhang M , Huang J , Tan X , et al. Common polymorphisms in the NFKBIA gene and cancer susceptibility: a meta‐analysis. Med Sci Monit. 2015;21:3186‐3196. 26488500 10.12659/MSM.895257 PMC4621165

[51] Shi D , Zhong Z , Xu R , et al. Association of ITGAX and ITGAM gene polymorphisms with susceptibility to IgA nephropathy. J Hum Genet. 2019;64:927‐935. 31227791 10.1038/s10038-019-0632-2

[52] Espinoza JL , Takami A , Onizuka M , et al. Recipient PTPN22‐1123 C/C genotype predicts acute graft‐versus‐host disease after HLA fully matched unrelated bone marrow transplantation for hematologic malignancies. Biol Blood Marrow Transplant. 2013;19:240‐246. 23025987 10.1016/j.bbmt.2012.09.014

[53] Alanazi M , Pathan AA , Shaik JP , et al. The C allele of a synonymous SNP (rs1805414, Ala284Ala) in PARP1 is a risk factor for susceptibility to breast cancer in Saudi patients. Asian Pac J Cancer Prev. 2013;14:3051‐3056. 23803078 10.7314/apjcp.2013.14.5.3051

[54] Jin J , Robeson H , Fagan P , et al. Association of PARP1‐specific polymorphisms and haplotypes with non‐small cell lung cancer subtypes. PLoS One. 2020;15:e0243509. 33284833 10.1371/journal.pone.0243509 PMC7721167

[55] Abecassis I , Sedgewick AJ , Romkes M , et al. PARP1 rs1805407 increases sensitivity to PARP1 inhibitors in cancer cells suggesting an improved therapeutic strategy. Sci Rep. 2019;9:3309. 30824778 10.1038/s41598-019-39542-2 PMC6397203

[56] Avitabile M , Lasorsa VA , Cantalupo S , et al. Association of PARP1 polymorphisms with response to chemotherapy in patients with high‐risk neuroblastoma. J Cell Mol Med. 2020;24:4072‐4081. 32103589 10.1111/jcmm.15058 PMC7171401

[57] Ramezani S , Sharafshah A , Mirzanejad L , et al. Association of PARP1 rs4653734, rs907187 and rs1136410 variants with breast cancer risk among Iranian women. Gene. 2019;712:143954. 31288058 10.1016/j.gene.2019.143954

[58] Mccarten KM , Nadel HR , Shulkin BL , et al. Imaging for diagnosis, staging and response assessment of Hodgkin lymphoma and non‐Hodgkin lymphoma. Pediatr Radiol. 2019;49:1545‐1564. 31620854 10.1007/s00247-019-04529-8

[59] Chen C , Song N , Dong Q , et al. Association of single‐nucleotide variants in the human leukocyte antigen and other loci with childhood Hodgkin lymphoma. JAMA Netw Open. 2022;5:e2225647. 35939300 10.1001/jamanetworkopen.2022.25647 PMC9361085

[60] Tanaka N , Mori S , Kiyotani K , et al. Genomic determinants impacting the clinical outcome of mogamulizumab treatment for adult T‐cell leukemia/lymphoma. Haematologica. 2022;107:2418‐2431. 35417939 10.3324/haematol.2021.280352 PMC9521232

[61] Chigrinova E , Mian M , Scandurra M , et al. Diffuse large B‐cell lymphoma with concordant bone marrow involvement has peculiar genomic profile and poor clinical outcome. Hematol Oncol. 2011;29:38‐41. 20635329 10.1002/hon.953

[62] Liu ZH , Zhang L , Jing FJ , et al. Genetic polymorphisms in NLRP3 inflammasome‐associated genes in patients with B‐cell non‐Hodgkin's lymphoma. J Inflamm Res. 2021;14:5687‐5697. 34754215 10.2147/JIR.S329090 PMC8570379

[63] Tibshirani R . Regression shrinkage and selection via the lasso. J R Stat Soc B. 1996;58:267‐288.

[64] Yang M , Ping L , Liu W , et al. Clinical characteristics and prognostic factors of primary extranodal classical Hodgkin lymphoma: a retrospective study. Hematology. 2019;24:413‐419. 30922173 10.1080/16078454.2019.1598678

[65] Mirili C , Paydas S , Kapukaya TK , et al. Systemic immune‐inflammation index predicting survival outcome in patients with classical Hodgkin lymphoma. Biomark Med. 2019;13:1565‐1575. 31631675 10.2217/bmm-2019-0303

[66] Zhao P , Zang L , Zhang X , et al. Novel prognostic scoring system for diffuse large B‐cell lymphoma. Oncol Lett. 2018;15:5325‐5332. 29552174 10.3892/ol.2018.7966 PMC5840739

[67] Sapkota S , Shaikh H . Non‐Hodgkin Lymphoma. StatPearls; 2023. 32644754

[68] Lindgren M , Samuelsson J , Nilsson L , et al. Genetic variation in IL28B (IFNL3) and response to interferon‐alpha treatment in myeloproliferative neoplasms. Eur J Haematol. 2018;100:419‐425. 29369421 10.1111/ejh.13034

[69] Kotenko SV , Gallagher G , Baurin VV , et al. IFN‐lambdas mediate antiviral protection through a distinct class II cytokine receptor complex. Nat Immunol. 2003;4:69‐77. 12483210 10.1038/ni875

[70] Sheppard P , Kindsvogel W , Xu W , et al. IL‐28, IL‐29 and their class II cytokine receptor IL‐28R. Nat Immunol. 2003;4:63‐68. 12469119 10.1038/ni873

[71] Paradowska‐Gorycka A , Wajda A , Stypinska B , et al. Interferons (IFN‐A/‐B/−G) genetic variants in patients with mixed connective tissue disease (MCTD). J Clin Med. 2019;8:2046. 31766529 10.3390/jcm8122046 PMC6947393

[72] Lan Q , Wang SS , Menashe I , et al. Genetic variation in Th1/Th2 pathway genes and risk of non‐Hodgkin lymphoma: a pooled analysis of three population‐based case‐control studies. Br J Haematol. 2011;153:341‐350. 21418175 10.1111/j.1365-2141.2010.08424.x PMC3075370

[73] Trinchieri G. Interleukin‐12: a proinflammatory cytokine with immunoregulatory functions that bridge innate resistance and antigen‐specific adaptive immunity. Annu Rev Immunol. 1995;13:251‐276. 7612223 10.1146/annurev.iy.13.040195.001343

[74] Mu S , Shi D , Ai L , et al. International prognostic index‐based immune prognostic model for diffuse large B‐cell lymphoma. Front Immunol. 2021;12:732006. 34745101 10.3389/fimmu.2021.732006 PMC8569825

