# Supplementary material for: The genetic polymorphisms of immune‐related genes contribute to the susceptibility and survival of lymphoma
Source: Cancer Med. 2023 Jun 16;12(14):14960–78. doi: 10.1002/cam4.6131 (PMC10417154; doi:10.1002/cam4.6131)
Supplement: Supplementary file 2 — Figure S2. [file CAM4-12-14960-s002.pdf]

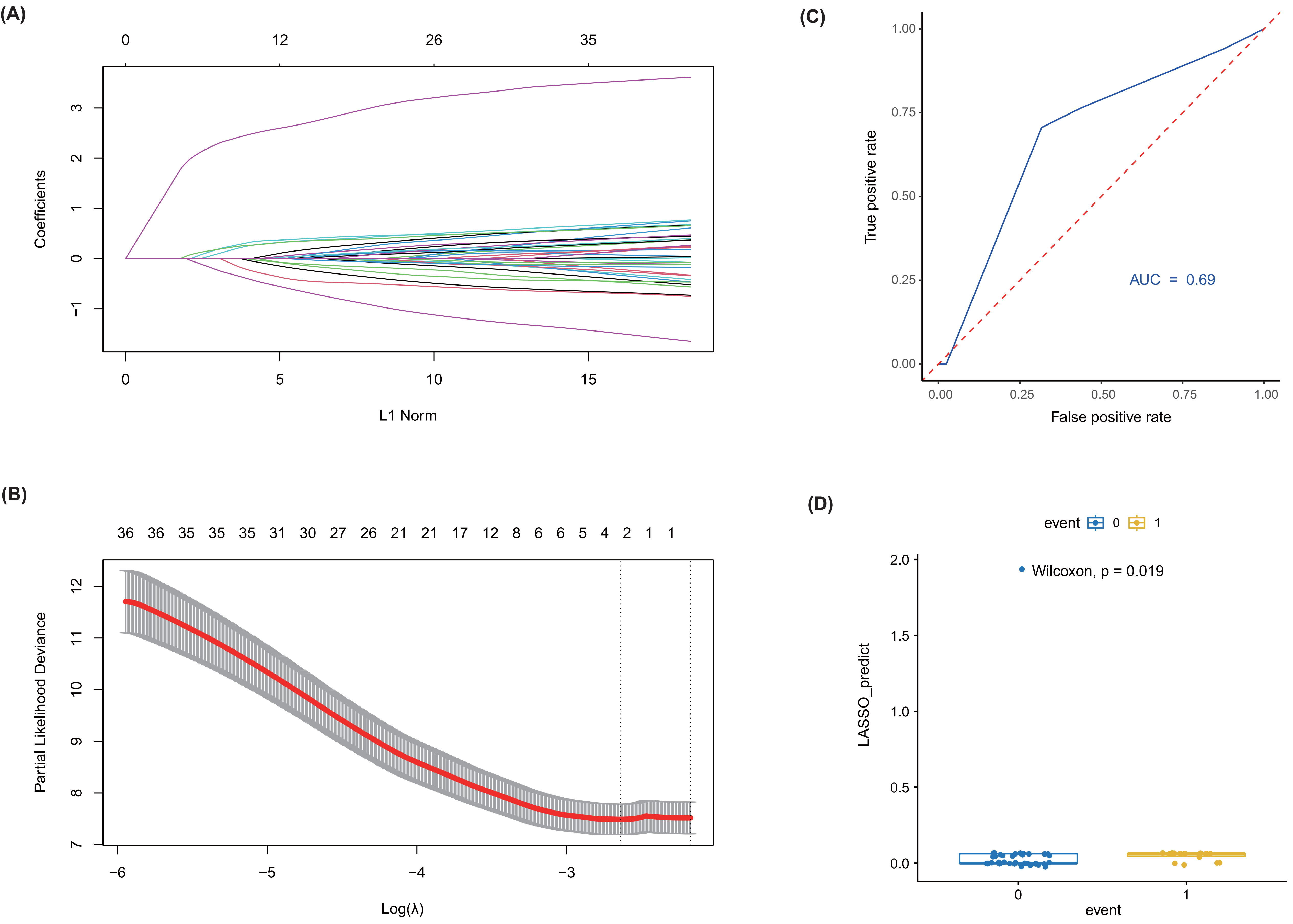

FIGURE S2. The selection of the variables by LASSO regression under additive model. (A). The variation characteristics of the coefficient of variables. (B). The selection process of the optimum value of the parameter λ in the LASSO regression model by ten fold cross-validation method. (C). ROC curve of additive model by LASSO regression. (D). The wilcoxon analyis between two survival states in test subsets under additive model.
